# Fabrication of Fe-Al Intermetallic Foams via Organic Compounds Assisted Sintering

**DOI:** 10.3390/ma8052217

**Published:** 2015-04-28

**Authors:** Krzysztof Karczewski, Wojciech Jerzy Stępniowski, Piotr Kaczor, Stanisław Jóźwiak

**Affiliations:** Department of Advanced Materials and Technologies, Military University of Technology, 2 Kaliskiego Str., Warsaw 00-908, Poland

**Keywords:** iron aluminides, self-propagating high-temperature synthesis, chemical compounds

## Abstract

The influence of the addition of organic compounds, such as palmitic acid and cholesteryl myristate, on the porous structure of Fe-Al intermetallics formation has been investigated in detail in this paper. It was found that additives have a significant effect on the final porosity of the obtained sinters. Formed gaseous products from combustion play the role of the foaming agent during Fe-Al intermetallic alloy sintering. The influence of these additives is also clearly noticeable in chemical composition changes of the final products through the increase of carbon content in the porous structure. This is attributed to the thermal decomposition, namely combustion, of the organic additives.

## 1. Introduction

Metal foams have potential for a variety of applications, such as ultra-light weight structural components, heat insulation, and energy absorbers. Commercially available metal foams are made mostly of aluminum alloys, which are manufactured using titanium hydride as a foaming agent [1,2,3]. However, the properties of metal foams fabricated from pure metals are often insufficient, especially if the foam has to work at a high temperature or in a corrosive environment. Intermetallic Fe-Al phase-based sintered porous materials constitute a promising group of modern engineering materials due to their attractive physical and mechanical properties, as well as the low cost of the raw elements [4,5,6,7,8,9]. This group of materials is known for their resistance towards corrosion and high temperature oxidation, as well as extraordinary mechanical properties at high temperatures [10,11,12,13].

Self-propagating high-temperature synthesis (SHS) assisted sintering of iron and aluminum elemental powders is connected by high intensity, high synthesis temperature, and limited possibilities of reaction control [14,15]. However, there are a number of parameters that affect the SHS reaction, such as: method of compact preparation, powder particle size and shape, reaction atmosphere, and heating rate [14,15,16]. Nevertheless, these parameters allow to control SHS only at a limited range, what translates into diffusion in the solid state. Another way to control the SHS process is doping of the starting materials with chemical compounds, which may have an impact on the produced material.

In this study, Fe and Al elemental powders, with and without additives, were fabricated by powder metallurgy. Two additives were used in the present study: palmitic acid (PA) and cholesteryl myristate (CM), which allowed the formation of highly porous materials thanks to their volumetric expansion during combustion.

## 2. Results and Discussion

**Figure 1:**
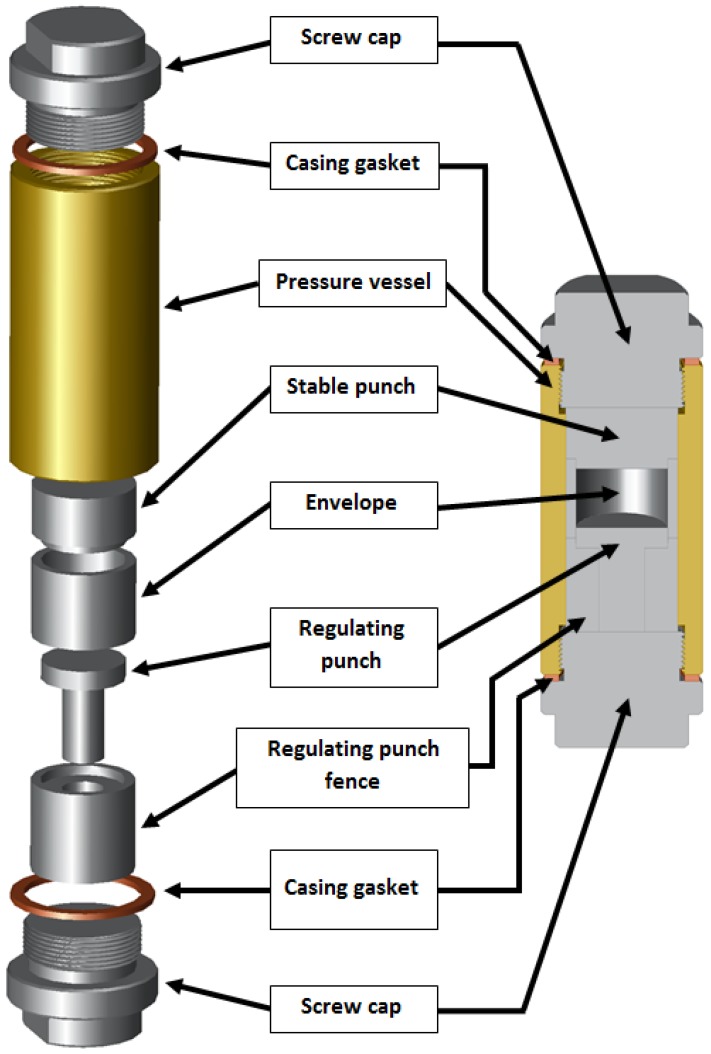
Scheme of the experimental reactor used in experiment.

**Figure 2:**
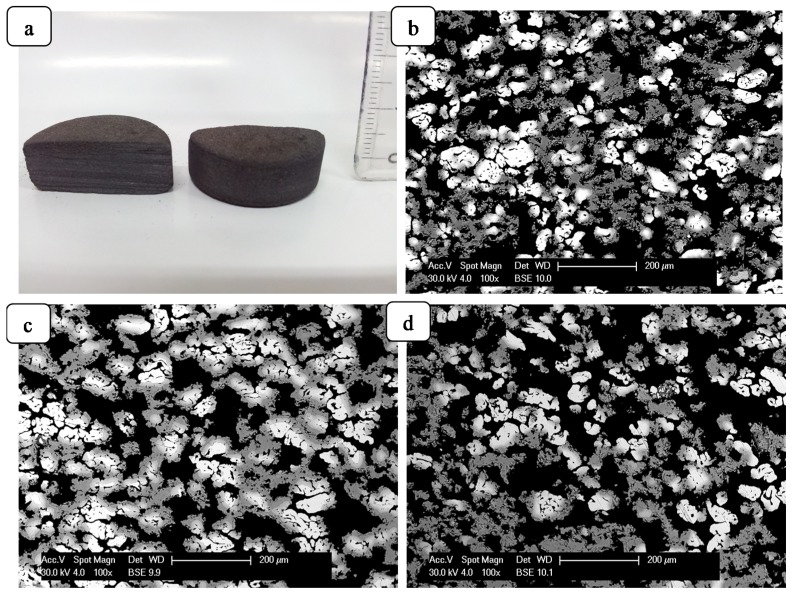
The exemplary of cross-sections of combustion-synthesized Fe-Al intermetallics (**a**); and microstructure of sinters: Fe–45Al (**b**); Fe–45Al–3% wt PA (**c**); and Fe–45Al–3% wt MC (**d**).

The synthesis of the porous materials was done with the application of a volume control environmental reactor (Figure 1; for further details see the Experimental Section). Figure 2 shows the exemplary cross-sections and porous microstructure of combustion-synthesized Fe-Al intermetallics with different amounts of chemical-compound additives. In order to determine the porosity of obtained sinters, the samples were observed with the use of optical microscopy (polished and unetched samples) and subsequently analyzed with an NIS-Elements image analyzer connected with Nikon MA200 microscope (Nikon, Chiyoda, Tokyo, Japan). Porosity measured by microscopic method is shown in the Table 1 and Figure 2. As expected, the final porosity of obtained sinters is the lowest for samples sintered without additives, and the highest for samples doped 3 wt% of the chemical compounds, regardless of whether palmitic acid or cholesteryl myristate was used. It should be noted that pores can be formed, not only by imbalance in mass transportation, but also by the pressing procedure, thus, either Kirkendall pores formed during sintering below the melting point of aluminum, or the pores formed through the rapid liquid Al reaction.

**Figure 3:**
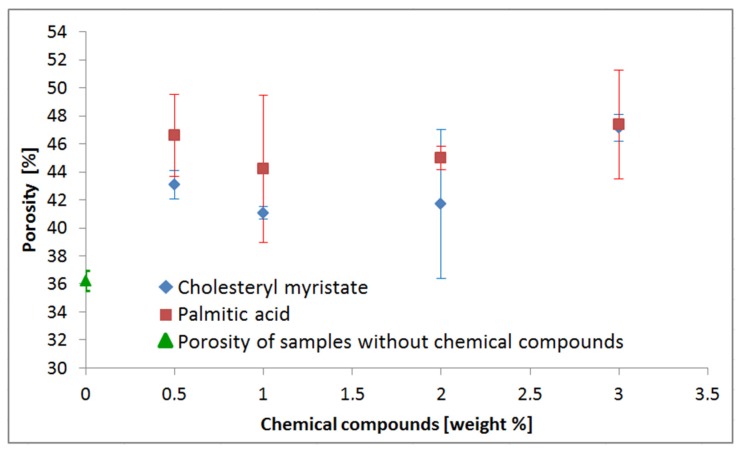
Influence of chemical-compound additive contents on the final porosity of sinters.

To investigate whether the porosity changes are statistically significant, ANOVA and MANOVA statistical tests were performed. It was found, that, despite relatively high values of standard deviation (Table 1, Figure 3), the influence of the added chemical-compound concentration on the porosity of the formed sinters was statistically significant (Table 2). ANOVA was performed separately for both chemical compounds and revealed statistically significant sinter porosity changes with the increase of the chemical-compound concentration. MANOVA confirmed the statistically significant influence of the chemical-compound concentration on the porosity of the formed sinters (Table 3), however, neither type of applied chemical compound (PA or CM), nor type of the compound-concentration interaction, has statistically influenced on the sinters porosity. Thus, the results indicate that the type of compound is not the most important (totally different molecular structure of the compound), but the amount of produced gases (water steam and carbon oxides) is the most important during the organic-compound-aided sintering.

**Table 1:** Porosity of obtained sinters.

Content of chemical compound (CM and PA) used in experiment (wt%)	Porosity (%)	
	Cholesteryl myristate (CM)	Palmitic acid (PA)
0	36.2 ± 0.7	
0.5	43.1 ± 1.0	46.6 ± 2.9
1	41.1 ± 0.4	44.2 ± 5.3
2	41.7 ± 5.3	45.0 ± 0.8
3	47.2 ± 1.0	47.4 ± 3.9

**Table 2:** Significance analysis of the influence of the chemical compound concentration on the porosity of the manufactured sinters (ANOVA).

Chemical compound	P < 0.05
Concentration of PA	+
Concentration of CM	+

**Table 3:** Significance analysis of the main factors (chemical compound concentration and type), and their mutual interactions on the porosity of the formed sinters (MANOVA).

Factor	P < 0.05	Interaction	P < 0.05
Concentration of the chemical compound	+	Concentration-type	−
Type of the chemical compound	−		

**Figure 4:**
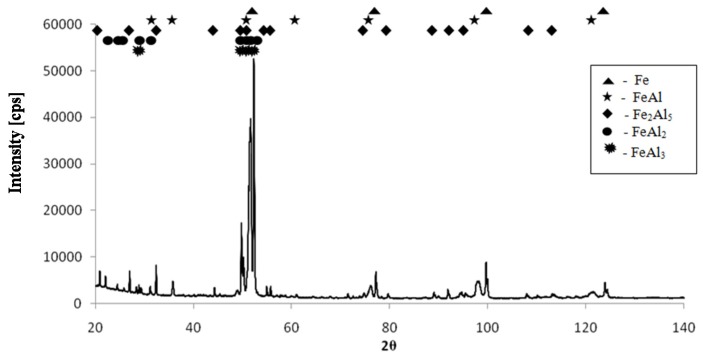
The exemplary XRD pattern of material after the sintering process (Fe–45Al–3 wt% MC).

**Figure 5:**
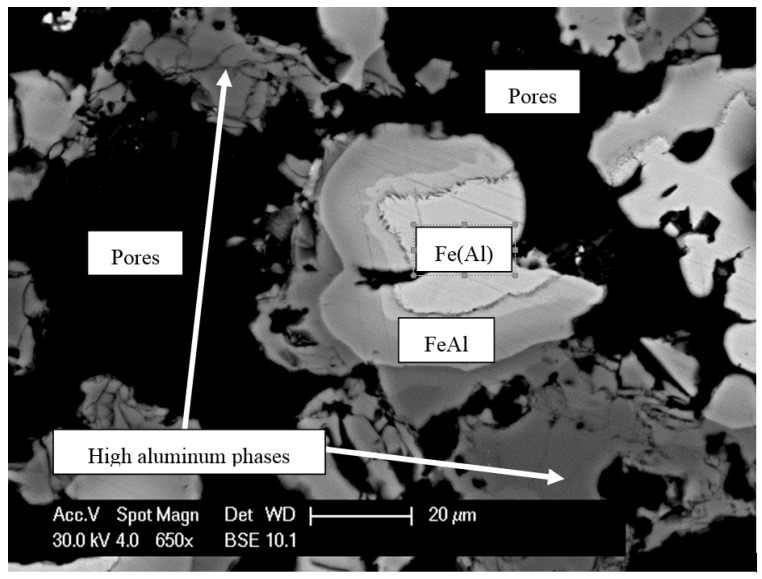
Result of EDS analysis in micro areas of material after the sintering process (Fe–45Al–3 wt% MC).

X-ray diffraction (XRD) analysis revealed that intermetallic phases from the Fe-Al equilibrium system can be found in all analyzed samples, including high-aluminum Fe_2_Al_5_, FeAl_3_, and FeAl_2_ phases (Figure 4). Scanning electron microscope (SEM) observations and energy-dispersive spectrometry (EDS) analysis confirm the heterogeneity of the obtained material structures (Figure 5).

**Figure 6:**
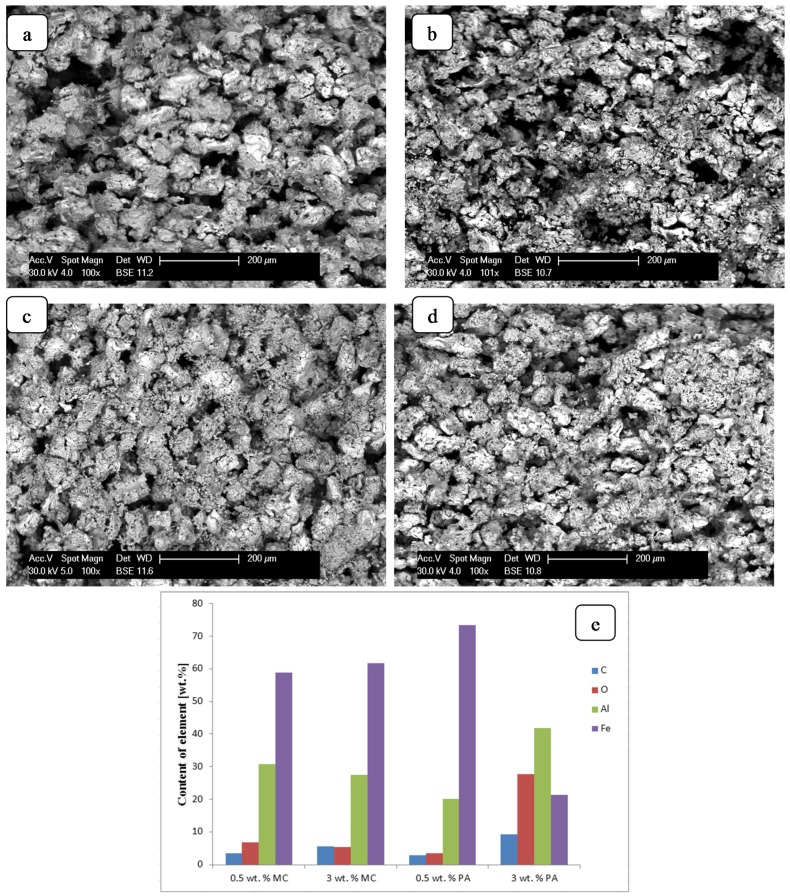
Microstructure of samples doped 0.5 wt% MC (**a**); 3 wt% MC (**b**); 0.5 wt% PA (**c**); 3 wt% PA (**d**); and EDS composition of obtained sinters (**e**).

EDS analysis of pore surfaces shows that with the increase of the added chemical compounds, the carbon content increases in the formed sinters (Figure 6d). However, the content of carbon measured by EDS analysis of samples can be overstated due to, for example, a contamination problem. In particular, the XRD patterns do not indicate the presence of carbon atoms or carbon phases (Figure 4). Thus, only amorphous, carbon-containing phases may be present in the formed material. However, one has to be aware that, for light elements, the EDS analyses should be treated only semi-quantitatively, due to the method’s poor accuracy with regards to carbon and oxygen.

The pores were formed due to the combustions of the chemical additives. These were chosen in such way to produce, during the process, only gaseous products, namely water steam and carbon oxides. Thus, during sintering, the combustion temperature of the above-mentioned chemical additives was achieved and the gaseous products were formed. Volumetric expansion of the gases occurred, accompanied by increased pressure, and the pores were formed. However, access to the air in the sinter was strongly limited. Therefore, during combustion, carbon dioxide and carbon monoxide were formed. Subsequently, carbon monoxide underwent disproportionation and carbon dioxide and carbon were formed. This carbon was adsorbed onto the surface of the metal, on the pore bottoms. This effect is observed as the increased carbon content in the chemical-composition analyses. Therefore, the only product on the combustion-aided Fe-Al intermetallic alloy sintering, is carbon, which is not disadvantageous either to iron or to aluminium and Fe-Al alloys. This is the major advantage of the proposed approach. For comparison, while one applies carbon containing inorganic salts, like carbonates (CaCO_3_, MgCO_3_, SrCO_3_, Li_2_CO_3_, BaCO_3_) [17,18], carbon oxides are formed as the foaming agents. However cations, in the form of metal oxides, are still present in the sinter and may form brittle phases with the sintered elements, which previously seemed to be unavoidable. This is the major disadvantage of Lost Carbonate Sintering (LCS). Moreover, via the LCS method, large irregular pores are obtained and their size and shape uniformity are poor [17]. The same problem occurs when researchers use metal hydrides as the foaming agent—hydrogen forms the pores, however, the metal remains in the sinter and may form other undesired phases in the whole sinter.

Comparable results of metal foaming were achieved by researchers when sodium chloride was added into the powder mixture prior to the sintering process [19,20,21]. However all the pores must be opened to flush out the salt after sintering, as chlorides are very corrosive, which seems to be the only disadvantage of this method. The researchers obtained porous Mg [19], Al-Mg alloy [20], or even Fe-Al intermetallic alloys [20].

Therefore, we were inspired to find, for our purposes, the results obtained when organic compounds were used as foaming agents. According to the literature data, stearic acid [22] or PMMA, polyethylene glycol, and stearic acid blends [23,24] are being used. According to the simple chemical calculations, one can obtain *ca*. 3.0 dm^3^ of gases at ambient conditions (carbon dioxide and water steam) from 1 g of stearic acid and a comparable amount, of 2.98 dm^3^, from 1 g of palmitic acid, which was used in the current research. However, the shorter the hydrocarbon chain, the easier the combustion is, and the lower the sintering temperature is, which is crucial for the foaming. The second applied compound, cholesteryl myristate, liquid crystal, provides 2.97 dm^3^, however, it does not have an aliphatic structure like PA or SA, but is an esther composed from cyclic parts. Therefore, it has a different molecular structure and liquid crystalline structure, providing better penetration. Thus, this was the starting point of our concept, supported by previously published premises [22,23,24].

## 3. Experimental Section 

The raw materials used in this study were: Fe powder, with an average particle size of 100 µm, (99.9% purity), Al powder, with an average particle size of <75 µm (99% purity), palmitic acid crystalline powder (PA), and cholesteryl myristate crystalline powder (CM). The following compositions were prepared: the reference composition (RC)—Fe-45Al (at%), RC + 0.5 wt% PA or CM, RC + 1 wt% PA or CM, RC + 2 wt% PA or CM and RC + 3 wt% PA or CM. Subsequently, the powder mixture was consolidated, by uniaxial cold pressing under 700 MPa pressure, into lasting cylindrical 25-mm diameter and 6-mm high pellets. The sintering process was conducted in the volume controlled environmental reactor (Figure 1), where the working space was controlled by a distance change between the spacer washer and the sample via screw rotation in an argon atmosphere at 700 °C for 3 hours. The structural analysis of the sinters was carried out with a Nikon MA200 optical microscope, integrated with NIS-Elements software applied to the porosity analysis. Additionally, chemical analysis was carried out with a PHILIPS XL30 (LaB_6_) SEM (Philips, Amsterdam, the Netherlands), integrated with an X-ray DX4i/EDAX microanalysis device. The identification of phases formed during the process was carried out by X-ray phase analysis.

Experiments were carried out three times at each point for statistical analyses, and the data were normalized for comparison. Statistical tests, typically applied in biochemistry, were applied in order to investigate the porosity of the sinters [25]. One-way analysis (ANOVA) was performed in order to investigate the statistical significance of the influence of each chemical compound concentration on the fabricated sinters’ porosity. Multi Way Analysis of Variance (MANOVA) was performed in order to estimate the significance of the main factors (chemical compound additive concentration, type of the chemical compound) on the sinters’ porosity, as well as their mutual interactions. To estimate the significant differences among parameters, the significance level was set at * P < 0.05. The calculations were made with Statistica 9.0 StatSoft, Inc. (Tulsa, OK, USA).

## 4. Conclusions

The manufacturing process of Fe-Al intermetallic foams, by using organic chemical compounds, such as palmitic acid and cholesteryl myristate as foaming agents enables the manufacturing of porous materials with a desired level of porosity. The self-propagating high-temperature synthesis of iron and aluminum powders, with the combustion of chemical additives, allowed the manufacturing of porous intermetallic structures with relatively regular and uniform morphology. Nevertheless, it seems very important to conduct further systemic studies of microstructures and the permeability of synthesized porous Fe-Al intermetallics.
